# Treatment failure in osteoporosis: who will experience a new Fracture? TAILOR a retrospective study

**DOI:** 10.1007/s40520-025-02972-2

**Published:** 2025-03-01

**Authors:** Ilaria Buondonno, Marco Di Stefano, Patrizia D’Amelio

**Affiliations:** 1 Department of Internal Medicine, Geriatric and Bone Disease Unit, University of Torino, 10126 Turin, Italy; 2 Department of Medicine, Service of Geriatric Medicine and Geriatric Rehabilitation, University of Lausanne Hospital (CHUV), Mont-Paisible 16, 1011 Lausanne, Switzerland

**Keywords:** Osteoporosis, Treatment failure, Inadequate responders, Fractures

## Abstract

**Background:**

Osteoporosis treatments reduce fracture risk but cannot fully eliminate it, and the concept of treatment failure (TF) or inadequate clinical response (ICR) remains debated.

**Aims:**

The TAILOR study aims to assess the prevalence of ICR and TF in osteoporotic women undergoing active drug treatment for postmenopausal osteoporosis.

**Methods:**

TAILOR is a retrospective study conducted in an Italian outpatient service. We included 415 patients with at least 12 months of treatment and up to 10 years, examining clinical characteristics predicting TF and ICR. TF was defined as the occurrence of two fragility fractures while on treatment or one fracture plus lack of variation BMD and ICR as the occurrence of a new osteoporotic fracture in treated patients according to previous literature.

**Results:**

Seventy-two patients experienced fractures during the follow-up, of those, 26 (36%) were classified as TF. The clinical characteristics of patients with fractures were similar to those without, except for a longer postmenopausal period and lower lumbar spine bone mineral density (BMD). Postmenopausal period was significantly longer in TF compared to ICR patients. However, no significant differences were found in baseline fractures, prescribed treatments, or fracture-free survival curves with age, postmenopausal period, BMD, and previous treatments. The clinical follow-up was longer in ICR and TF patients.

**Discussion:**

TAILOR shows a higher prevalence of ICR and TF (17.3%) compared to randomized controlled trials and real-world data, with 36% of fractures during follow-up classified as TF. Clinician decisions often led to changes in antiosteoporosis treatment, particularly in TF cases, though TF diagnosis was rarely cited explicitly in medical records.

**Conclusion:**

TAILOR emphasizes that common clinical factors do not reliably predict ICR and TF. The findings highlight the complexity of determining an algorithm for the best treatment approach to prevent TF and ICR.

## Introduction

Fragility fractures represent a significant clinical burden in an increasingly older population. Older patients suffering from femoral fractures have a high mortality rate within a year (15–25%) and a substantial likelihood of becoming dependent (50%). The costs associated with incident and prior fragility fractures in 2019 in was estimated at 57 billion €/year in Europe [1]. In the US Singer and colleagues reported the mean annual healthcare costs among patients with fragility fracture were $44,311 (± $67,427) and even higher if diagnosed after hospitalization [2] The appearance of a second fractures exacerbate patients' health issues and increase costs [3, 4]. The financial burden imposed by second fractures on the United States healthcare system has been calculated at 2 billion USD/year [5].

The efficacy of osteoporosis treatment lies in reducing the fracture risk. Drugs approved for osteoporosis can decrease the risk of fractures by 30–70% for vertebral fractures, 40–50% for hip fractures, and 15–20% for non-vertebral fractures [6, 7].. Treatment efficacy is assumed by a significant reduction in fracture risk supported by a stability or an increase in bone mineral density (BMD) and a decrease in markers of bone resorption. The question if a new fracture or bone loss may be considered a treatment failure is highly debated, a position paper of the International Osteoporosis Foundation described the treatment failure (TF) based on the occurrence of two fractures or one fracture plus lack of variation in BMD, markers of bone resorption or both while on treatment [8]. According to this definition patients sustaining a new osteoporotic fracture during treatment cannot automatically be classified as TF, for these patients the definition of “inadequate clinical treatment response” (ICR) has been proposed [9]. Although the proportion of ICR can be estimated from the registration trials of the anti-osteoporosis medications as fluctuating between 2.1% and 18.1% over 3–5 years depending on the severity of the disease [10–16], there are few studies on nonresponse in clinical practice showing an ICR incidence ranging between 8.9 and 9.5% per year [9, 17]. Patients in the real world differ from those included in RCTs, often being older, having comorbidities, possessing less information, and displaying lower motivation, with a less strict follow-up. These factors, coupled with limited compliance to anti-osteoporotic treatment, have been cited to explain ICR and treatment failure in clinical practice [18]. However, accurately predicting patients' responses to treatment to assist physicians in selecting the best course of action remains a challenge. Following an ICR or a TF, clinicians face the challenge of deciding whether to start/stop or change treatment. This decision relies entirely on their medical experience, and clinical practice is not uniform.

Studying the factors that contribute to ICR and TF in clinical practice is crucial. The accurate and early identification of patients who will not benefit from first-line drugs can prevent unsuccessful treatment and reduce healthcare costs.

Therefore, the primary objective of the TAILOR study is to assess the prevalence of ICR and TF and analyze the clinical characteristics that predict treatment failure in a clinical setting.

## Materials and methods

TAILOR is a retrospective study carried out on a cohort of osteoporotic women coming to the outpatients’ service of the Unit of Geriatrics and Bone Metabolic Disease in Torino (Italy) and treated with anti-resorptive or anabolic drugs for postmenopausal osteoporosis. We included in the analyses all the women evaluated in the out-patients service of our unit between 01.01.2009 and 31.12.2019.

Inclusion criteria were diagnosis of postmenopausal osteoporosis actively treated for at least 12 months and up to 10 years with drugs active on bone turnover and with at least one follow-up visit in the 10-year period considered, medical records available and complete.

To exclude possible bias male subjects, patients with secondary osteoporosis and with medical records incomplete were excluded.

The ethical committee approved the protocol (approval number 00418/2020, date 01.10.2020).

TF has been defined according to the position paper of the IOF [8] as the occurrence of two fragility fractures while on treatment or one fracture plus lack of variation BMD over, at least, one year. Bone resorption markers changes has not been considered as these markers were not routinely measured in the included cohort according to the Italian guidelines [19]. ICR has been defined as the occurrence of a new osteoporotic fracture in treated patients [9]. BMD was measured in the center for all the patients with a Hologic QDR 4500 X-ray densitometer. According to the guidelines, secondary osteoporosis was ruled out through appropriate laboratory investigations before classifying a patient as ICR or TF. Fragility fractures were defined as a fracture occurring after a fall from the standing position or a mechanical effort without fall according to standard clinical practice. Vertebral fractures were diagnosed by X-ray or DXA morphometry, for non-vertebral fractures a hospital discharge letter or the physician report was considered as evidence of fracture. Fractures of the skull, face, cervical spine, fingers, and toes have not be considered as TF according to the IOF position paper [8].

Patients were classified as adequate responders (ARs) to antiresorptive after at least 60 months on treatment without incident fractures according with Diez-Perez et al. [20], ARs to teriparatide where defined as patients not experiencing new fractures during the 24 months treatment period.

For each patients the following information have been recorded: type of active treatment (SERMs, oral bisphosphonates, iv bisphosphonates, denosumab or teriparatide), treatment with calcium and vitamin D in association to active treatment, age, postmenopausal period, presence and site of fragility fractures, BMD values. This information has been collected for each visit at center, the decision of the physician expert in osteoporosis management during the follow-up visit regarding treatment (continue/stop/change treatment) has also been recorded.

### Statistical analyses

The sample size calculation was conducted using an estimated medium effect size (*f* = 0.25), an alpha level of 0.05, and a power of 0.95 using the software G*power. According to statistical computing, a sample size of *n* = 400 is required to estimate TF incidence.

Characteristics of patients experiencing TF were compared to ARs by means of ANOVA or χ2 test for the analyzed variables. To estimate the survival to the first fracture at follow-up we build up a Kaplan-Meyer curve and used a Cox regression model to estimate the effects of variables different between Ars, ICR and TF on the risk of fracture.

## Results

We screened 2205 medical records of patients consulting between 01.01.2009 and 31.12.2019, of those 228 (10%) were excluded for incomplete data, 799 (36%) for violation of inclusion criteria (206 patients were male and 365 were affected by secondary osteoporosis), 763 (35%) did not undergo follow-up for a minimum of 60 months. We included in the study medical records from 415 patients affected by postmenopausal osteoporosis.

### Patients’ general characteristics

The mean age of included patients was 65 ± 9 years, with a mean postmenopausal period of 16 ± 9 year; at the first visit 174 patients (41.9%) have already experienced one or more osteoporotic fractures. Seventeen patients experienced a femoral fracture, 48 a vertebral fracture, 10 a non-vertebral, non-femoral osteoporotic fracture, 92 presented at the first visit with multiple vertebral fractures, 2 with both a vertebral and a femoral fracture and 4 with a vertebral and another osteoporotic fracture. During the first visit 236 patients (56.9%) received a prescription for an active anti-osteoporotic treatment associated to calcium and vitamin D, while 179 received only calcium 1–1.2 g per day and vitamin D 800 IU per day, amongst patients receiving an active treatment for osteoporosis 166 (70.3%) have already experienced a fracture. Amongst patients treated with active anti-osteoporotic treatment, 127 were treated with oral bisphosphonates, 35 with strontium ranelate, 21 with zoledronates, 37 with teriparatide, 15 with denosumab and 1 with raloxifene. Among the 37 patients treated with teriparatide, 30 received this drug as a first-line treatment (in accordance with Italian reimbursement criteria) due to the presence of multiple vertebral fractures at diagnosis, while 7 received the drug after experiencing an osteoporotic fracture following at least one year of treatment with oral bisphosphonates.

The mean follow-up period was 2479 ± 617 days.

### Fractures during treatment and treatment failure

Seventy-two patients treated with active treatment for at least one year experienced a fracture during the follow-up, amongst those 26 (36%) can be classified as TF according to literature [8].

Patients experiencing fractures during follow-up had clinical characteristics comparable to those that did not experience fractures except for a longer postmenopausal period and a lower BMD at lumbar spine. Postmenopausal period was also significantly longer in patients classified as TF as respect to ICR (Table 1), whereas there was not significant difference in the type of fractures present at baseline between the three groups of patients (data not shown), nor in the treatment prescribed (Table 1). The clinical follow-up was longer in ICR and TF patients. As regards calcium and vitamin D supplements we found data on medical records for 349 patients clearly stating if treatment with calcium, vitamin D, both or none was started, overall, 250 patients received treatment with calcium (1–1.2 g per day) and/or vitamin D 800 UI per day (71.6%); the prescription of supplements was not significantly different amongst ARs, ICR and TF (Table 1).

**Table 1.** Clinical characteristics of patients according to ICR and TF during follow-up. Mean with SD and p values of the one-way ANOVA with the post-hoc Bonferroni correction are shown

	AR (343)	ICR (46)	TF (26)	*p*
Age (Years)	65±9	64±8	68±8	0.245
Post-menopausal period (Years)	16±9	12±8	20±11*	0.038
Fractured at first visit number of patients (%)	151 (44%)	14 (30%)	13 (50%)	0.163
Prescription of calcium and vitamin D, number of patients (%)	215 (72.1%)	25 (73.5%)	10 (58.8%)	0.479
Total femur T-score (SD)	−2.6±0.6	−2.8±0.6	−2.2±0.7	0.209
BMD spine T-score (SD)	−3.0±0.630	−3.7±0.55	−2.8±0.548	0.042
Follow-up (months)	80±18	95±5	92±4	<0.001
Patients treated with oral Bisphosphates (%)	30%	37%	26%	0.120
Patients treated with zoledronate (%)	4%	5%	6%	
Patients treated with Teriparatide (%)	17%	0.1%%	11%	
Patients treated with Denosumab (%)	0.1%	0.03%	11%	
Patients treated with Strontium Ranelate (%)	0.1%	0.1%	31%	
Patients treated with SERMs (%)	0%	0.03%	0%	

*Post hoc Bonferroni test showing significant differences between patients experiencing a new fracture during follow up an patients classified as TF (*p*=0.034).

**Fig. 1:**
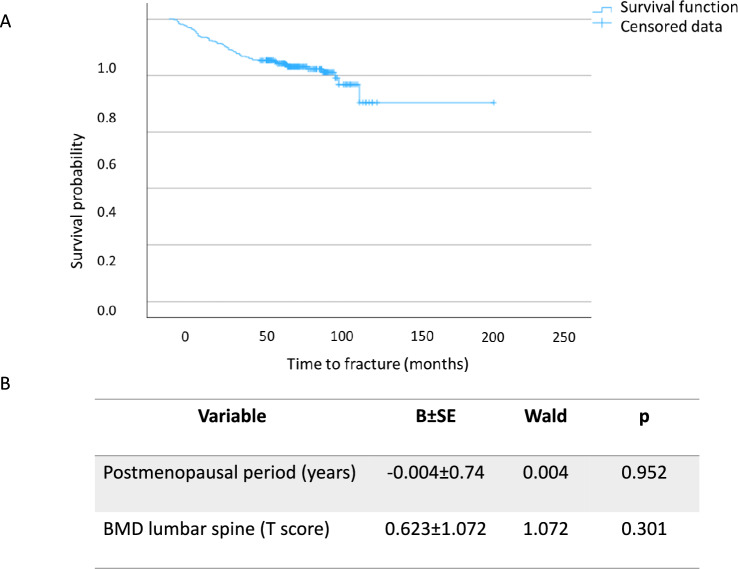
Follow-up free of fractures. Kaplan–Meier curve for the appearance of the first fracture at follow-up (panel **A**). The table shows influence of postmenopausal period an BMD on the appearance of fractures during follow-up (Cox regression, panel **B**)

The mean follow-up until the first fracture was 27 ± 12 months, the introduction on postmenopausal period and lumbar T-score as co-variables did not significantly affect the survival curve (Fig. 1).

During the follow-up the physician changed treatment in 204 patients (49%), in ICR patients this percentage raised up to 74% and up to 88.5% in patients classified as TF. Doctors considered the fracture during follow-up as a significant reason to change treatment (*p* < 0.001), however the diagnosis of TF as respect to ICR does not significantly influence their decision (p = 0.227), in any of the analyzed medical record TF was explicitly cited.

## Discussion

The TAILOR study addresses a critical aspect of clinical practice by investigating the prevalence and factors contributing to ICR and TF in patients affected by postmenopausal osteoporosis examined in a center for bone diseases in Italy.

The identification of patients who may not benefit from first-line drugs is paramount for optimizing treatment strategies in clinical practice. ICR and TF represent challenges that, if not addressed early, can lead to prolonged suffering for patients, increased healthcare costs, and potential long-term complications. Understanding the prevalence of ICR and TF is crucial for refining treatment protocols and improving patient outcomes. The prevalence of these condition has been mainly derived from RCT for different antiosteoporosis drugs, however factors influencing ICR and TF and the challenge of envisioning this condition has been rarely investigated in clinical practice [4, 9, 17], moreover the clinical decision following these events has not been standardized.

Patients referred to the center for the treatment of osteoporosis are complex and frequently had already experienced a fracture, and a significant proportion of those received an active treatment already at the first visit, highlighting the challenges in preventing subsequent fractures despite ongoing therapy.

We show a prevalence of 17.3% of ICR and TF, higher than the prevalence observed in RCT and in real world studies [9, 17]. Notably, 36% of the fractures occurred during follow-up can be classified as TF according to literature definitions [8], this high prevalence can be attributed to the high risk profile of those patients. Patients experiencing ICR or TF had a longer postmenopausal period and lower BMD, however, differently from other studies [9, 17], we cannot found significant difference in calcium and/or vitamin D prescription in these subjects. When analyzed as risk factors for ICR and TF both postmenopausal period and BMD did not significantly affect the risk. The clinical follow-up was significantly longer in patients experiencing ICR and TF, this is not surprising and has already be reported [9], this is probably due to the fact that patients experiencing a fracture during treatment are more likely to come back at center for further controls. Differently from others real world studies [4, 17] we did not found significant difference in patients age and in the presence of multiple vertebral fractures, on the contrary the type of the first fracture was not significantly different between Ars, ICR and TF. It is important to note, however, that common clinical factors such as comorbidities, lifestyle factors, or educational background were not included in our analysis. This limitation may affect the interpretation of these findings and warrants further investigation in future studies.

The variety of treatments administered, including oral bisphosphonates, strontium ranelate, zoledronates, teriparatide, denosumab, and raloxifene, reflects the diverse therapeutic approaches employed in clinical practice and offers a real-world diving. In our cohort 18.9% of the patients receiving teriparatide as first drug prescription had sustained a fracture while undergoing treatment with an antiresorptive prior to initiating this drug. This finding reflects the criteria for teriparatide prescription in Italy, which prioritize patients with severe osteoporosis and a history of multiple fractures or fractures during prior treatment. It is plausible that this subgroup, characterized by a higher fracture risk, demonstrated better adherence to therapy compared to patients treated with bisphosphonates, as severe disease often necessitates closer monitoring and tailored treatment strategies.

As regards clinician decision facing an ICR it is interesting to note that in 74% of cases the expert doctor proposed to change the antiosteoporosis treatment, the same behavior raise up to 88.5% in patients retrospectively classified as TF. While fractures during follow-up influenced treatment decisions significantly, the diagnosis of TF did not appear to be a decisive factor, with physicians rarely explicitly citing TF in medical records.

TAILOR faces some limits and in particular the analysis of patients coming to a single centre and exclusion of men and secondary osteoporosis can limit the generalization of our findings. However being monocentric can also be seen as a strength of the study, as the clinical practice amongst doctors working in the same centre is uniform as demonstrated by the elevated prescription of calcium and vitamin D supplements, this limits the bias due to increased recurrence of fractures due to malpractice [9, 21]. Moreover, we do not have an objective measure of compliance or adherence to treatment, except for the patients' self-reports. However, this limitation is intrinsic to the study design and, in our opinion, cannot be overcome. Nevertheless, our analysis revealed no significant differences in the incidence of TF and ICR between patients receiving oral, sub-cutaneous or intravenous anti-osteoporotic treatments, despite the well-documented association between reduced compliance to osteoporosis medications and increased fracture risk. Moreover, data on fracture risk assessment using an established international tool, such as FRAX or other validated models, were not available, which could have provided a more standardized interpretation of fracture risk and its relationship with treatment modalities.

The results from TAILOR appears to be immediately generalizable to clinical practice. The main strength of TAILOR is its observational design that allow us to dive into every day clinical practice.

## Conclusions

In conclusion according to our data general recommendation from the TAILOR study is to closely follow-up the patients especially those with postmenopausal longer than 16 years and spine BMD lower than −2.9.

The TAILOR study highlights the ongoing challenges in managing postmenopausal osteoporosis effectively. The need for early identification of patients at risk for TF and ICR and the importance of adapting treatment strategies to individual patient characteristics are evident. The study's findings underscore the multifaceted nature of osteoporosis and the importance of a personalized, dynamic approach to treatment in clinical practice. However, factors commonly evaluated in clinical practice for the treatment choice as age, postmenopausal period, BMD, previous anti-osteoporotic treatment and presence and site of fractures does not appear to be significant predictors of ICR and TF, hence we cannot conclude by suggesting a significant algorithm to the best treatment approach to prevent TF and ICR.

## Data Availability

Data are available upon reasonable request to the corresponding author.
